# Partial Deletion of the Sulfate Transporter *SLC13A1* Is Associated with an Osteochondrodysplasia in the Miniature Poodle Breed

**DOI:** 10.1371/journal.pone.0051917

**Published:** 2012-12-26

**Authors:** Mark W. Neff, John S. Beck, Julie M. Koeman, Elissa Boguslawski, Lisa Kefene, Andrew Borgman, Alison L. Ruhe

**Affiliations:** 1 Laboratory of Neurogenetics and Canine Behavior, Van Andel Research Institute, Grand Rapids, Michigan, United States of America; 2 Program for Canine Health and Performance, Translational Genomics Research Institute, Phoenix, Arizona, United States of America; Indiana University School of Medicine, United States of America

## Abstract

A crippling dwarfism was first described in the Miniature Poodle in Great Britain in 1956. Here, we resolve the genetic basis of this recessively inherited disorder. A case-control analysis (8∶8) of genotype data from 173 k SNPs revealed a single associated locus on *CFA14* (P_raw_ <10^–8^). All affected dogs were homozygous for an ancestral haplotype consistent with a founder effect and an identical-by-descent mutation. Systematic failure of nine, nearly contiguous SNPs, was observed solely in affected dogs, suggesting a deletion was the causal mutation. A 130-kb deletion was confirmed both by fluorescence *in situ* hybridization (FISH) analysis and by cloning the physical breakpoints. The mutation was perfectly associated in all cases and obligate heterozygotes. The deletion ablated all but the first exon of *SLC13A1*, a sodium/sulfate symporter responsible for regulating serum levels of inorganic sulfate. Our results corroborate earlier findings from an *Slc13a1* mouse knockout, which resulted in hyposulfatemia and syndromic defects. Interestingly, the metabolic disorder in Miniature Poodles appears to share more clinical signs with a spectrum of human disorders caused by *SLC26A2* than with the mouse *Slc13a1* model. *SLC26A2* is the primary sodium-independent sulfate transporter in cartilage and bone and is important for the sulfation of proteoglycans such as aggregan. We propose that disruption of *SLC13A1* in the dog similarly causes undersulfation of proteoglycans in the extracellular matrix (ECM), which impacts the conversion of cartilage to bone. A co-dominant DNA test of the deletion was developed to enable breeders to avoid producing affected dogs and to selectively eliminate the mutation from the gene pool.

## Introduction

**Figure 1:**
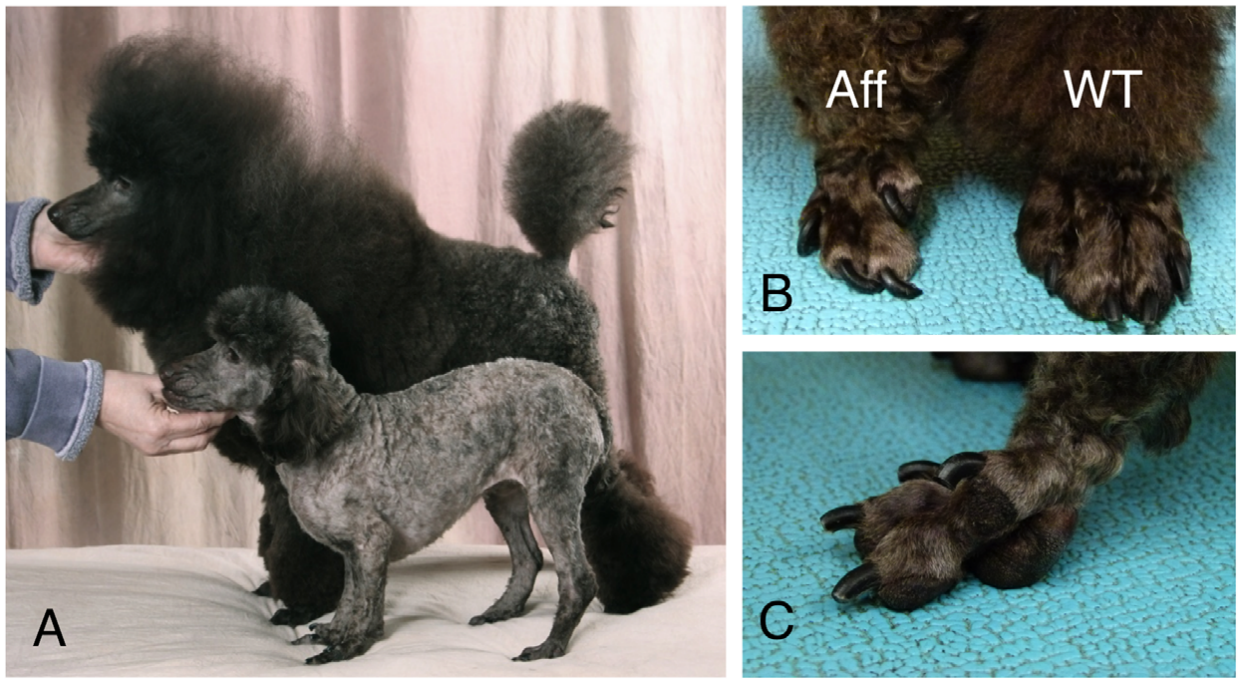
Gross presentation of Miniature Poodle osteochondrodysplasia. (A) An affected dog (foreground) shows dwarfism relative to an age-matched normal (wildtype) dog (background). (B) The misshapen paw of an affected dog (Aff) relative to that of a wildtype (WT) dog. (C) Close-up of the affected foot, which is similar to talipes varus, or clubfoot, a distinguishing feature of human osteochondrodsyplasias.

The osteochondrodysplasias represent a broad group of cartilage and bone disorders stemming from structural, metabolic, and endocrinological causes. An osteochondrodysplasia was described in the Miniature Poodle breed nearly 60 years ago [1], though the defect remains unresolved molecularly. The disorder is grossly characterized by stunted growth and abnormal locomotion, first observable at three weeks of age. Affected pups soon exhibit abducted hind limbs, enlarged joints, dorsoventral flattening of the rib cage, shortened and bent long bones, undershot jaws, and elongated and misshapen paws that resemble clubfoot [1]–[7]. Radiographic stippling is found at the epiphyses, reflecting aberrant conversion of cartilage to bone. The vertebrae are often beaked at their ventral surface, a clinical hallmark of several human skeletal dysplasias. The stiffness of joints that is profound in young affected dogs lessens with maturation, but mobility remains restricted and arthritis is a common sequelae. **Figure 1** shows gross characteristics of Miniature Poodle osteochondrodysplasia.

The Miniature Poodle defect has been compared to human disorders pseudoachondroplastic dysplasia (PSACH; OMIM#177170) and multiple epiphyseal dysplasia (MED; OMIM#132400) [2], [7]. Human PSACH results from mutations in a single structural gene, *COMP*, which encodes the cartilage oligomatrix protein, a major organizing protein of the extracellular matrix (ECM) of cartilage. The causal mutations in *COMP* act in a dominant negative manner. The Miniature Poodle disorder shows an autosomal recessive pattern of inheritance [8].

Human MED is phenotypically milder than PSACH and is also more genetically heterogeneous. The overlap of these two diseases has recently been characterized [9]. MED has been found to stem from mutations in five structural genes (*COMP*, *COL9A1*, *COL9A2*, *COL9A3*, and *MATN3*) and in a sixth, metabolic gene (*SLC26A2*, a sulfate ion transporter). The phenotypic spectrum of MED overlaps with the Miniature Poodle disorder, but there are notable differences. Human MED primarily affects the epiphyses of the long bones and minimally affects overall size and stature. In contrast, the Miniature Poodle disorder affects bones of the axial skeleton (*i.e.*, rib cage and vertebral column) and of the appendicular skeleton, and it includes obvious stunted growth (dwarfism). Anecdotal reports from owners also note that most affected dogs have protruding mandibles. Given these discrepancies, we refer to the Miniature Poodle disorder as an osteochondrodysplasia, the molecular basis of which may ultimately decide the appropriate classification.

We undertook this project with the aim of translating a mutation discovery into a DNA test capable of guiding breeding decisions. Because Miniature Poodle osteochondrodysplasia shares overlapping clinical features with genetically heterogeneous human skeletal dysplasias, we favored unbiased genome-wide mapping rather than a targeted candidate gene approach. Here, we report the mapping of the Miniature Poodle disorder to a single locus in the dog genome, consistent with an apparent Mendelian inheritance. We identified the presumptively causal mutation, and we leveraged this information to design, develop, and validate a co-dominant DNA test capable of discerning all three genotypes. The causative gene, *SLC13A1*, implicates aberrant sulfate metabolism and transport in this disorder. We found that some of the defects observed in affected Miniature Poodles recapitulate those found in an *Slc13a1* knockout mouse [10]. The Miniature Poodle osteochondrodysplasia appears to share even more features with human disorders resulting from loss-of-function mutations in *SLC26A2*, a member of a distinct family of anion transporter genes [11]. We discuss the osteochondrodysplasia in the context of a sulfate metabolic disorder that may be more broadly syndromic, involving other phenotypes not yet documented clinically.

## Results

### Association of Miniature Poodle Osteochondrodysplasia with a Single Genomic Locus

**Figure 2:**
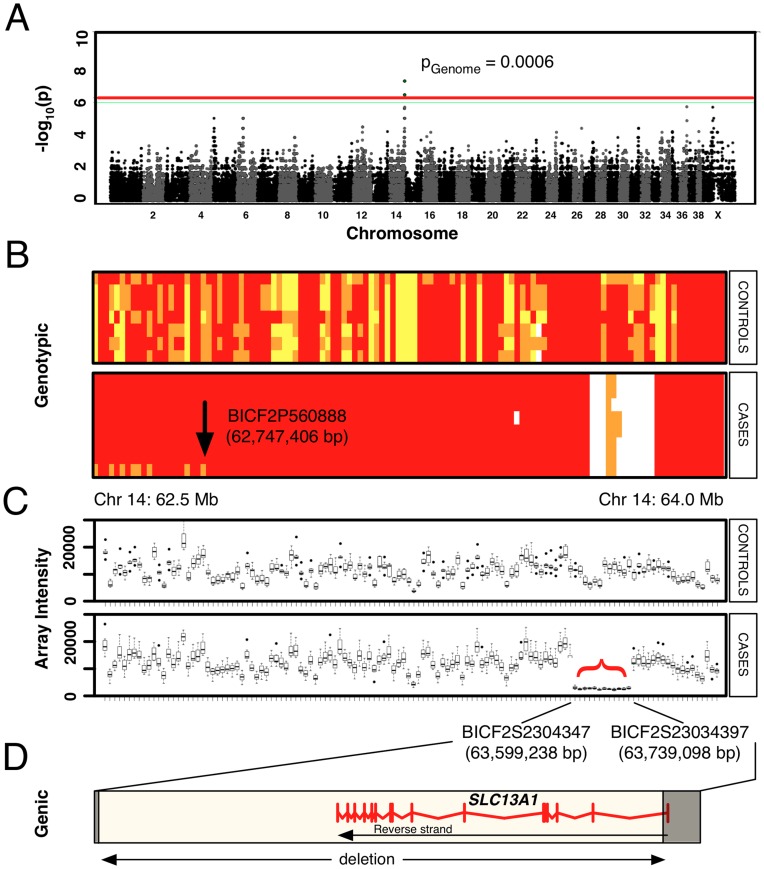
Genetic mapping of the osteochondrodysplasia locus. (A) Manhattan plot of genome-wide results displaying a statistically significant peak of association on the terminal end of *CFA14*. Plotted *p*-values were calculated by Fisher’s Exact Test. The p-values for the six most strongly associated SNPs (overlapping on the graph), adjusted by permutation, were p = 0.0006. (B) Genotypic pattern of SNPs across the interval of interest on *CFA14* (62.5–64.0 Mb). Red, major allele in cases; orange, heterozygous SNP genotypes; yellow, homozygous minor allele in cases. Genotype data are shown horizontally for each of seven controls and eight cases. The arrow notes an historical crossover that bounds the ancestral haplotype shared among cases, upon which the founder mutation is predicted to have occurred. Nine SNPs systematically failed in the case samples (white block with orange), suggesting a large deletion. (C) Raw fluorescence intensity readings from the SNP array for loci across the region of interest (62.5–64.0 Mb). Although several SNPs were initially scored heterozygous (orange, in B), the raw intensity data shows systematic failure in cases for all 12 SNPs in this interval (red bracket), relative to controls. (D) The potential deletion was bounded by two SNPs that were scored in cases. The putative deletion includes a single gene, *SLC13A1*, drawn to scale, with exon structure in red. The location of physical breakpoints that were subsequently cloned are shown as opaque boxes.

SNP genotyping was performed on 8 cases and 8 controls. The average call rate was greater than 98% for each dog analyzed. Genome-wide association revealed a single locus on the terminal end of chromosome 14 (*CFA14*) associated with the defect (P_raw_<10^–8^; P_Genome_ = 0.006) (**Figure 2A**). The mapped locus revealed a single ancestral haplotype that was homozygous in all cases (**Figure 2B**), consistent with recessive inheritance of a founder mutation. The extended causative haplotype (1.19 Mb) included six genes, which have been listed in the supporting information (**Document S1**).

### Genotypic Evidence of a Deletion in the Mapped Interval

SNP genotypes across the mapped region revealed systematic failure of 12 contiguous SNPs (**Figure 2B and C**). The SNP pattern suggested the presence of a spontaneous deletion. The SNP markers spanned an interval 139,860 bp long (Chr14∶63,599,238–63,739,098). The locus is 200 kb from the terminal end of *CFA14*. The telomeric ends of chromosomes are known to be hyper-recombinogenic in mammals, including *C. familiaris* [12]. If null SNPs were indeed reflective of a deletion, 14 of 15 exons of *SLC13A1* (alias *NAS1*) would be removed, truncating 95% of the peptide (**Figure 2D**). SLC13A1 is a sodium-dependent sulfate transporter that acts as a master regulator of serum sulfate levels [13]. Sulfation of proteoglycans in the ECM of cartilage is important for skeletal development [14].

### Cytogenetic Analysis Confirms a Deletion Mutation

**Figure 3:**
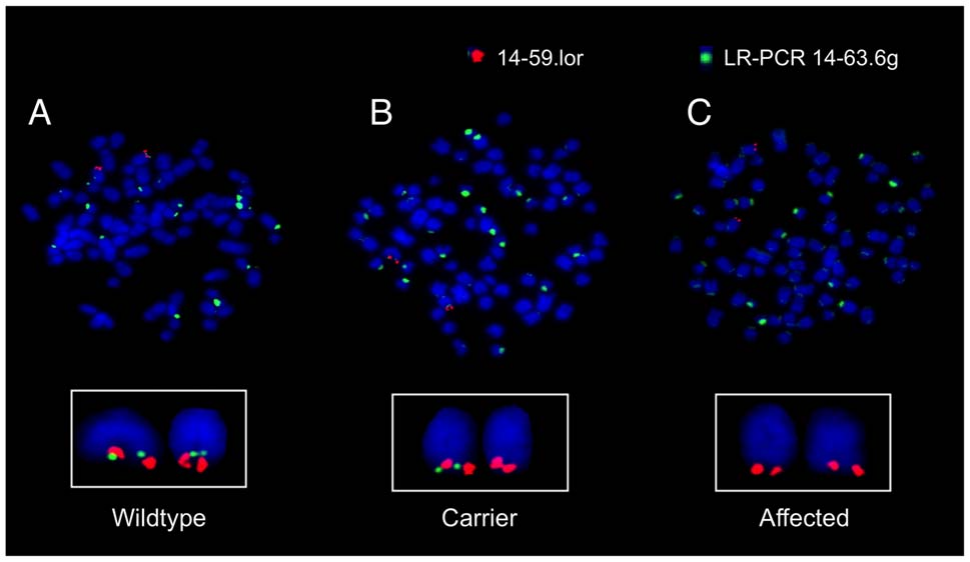
Cytogenetic confirmation of a deletion on chromosome 14 by *FISH*. Dual-color FISH probe results are shown for representative metaphase spreads obtained from (A) wildtype, (B) obligate carrier, and (C) affected Miniature Poodle dogs, respectively. The green LR-PCR 14–63.6 g FISH signal is within the deleted region and shows some centromeric background staining. The red BAC probe 14–59.1or signal distinguishes *CFA14* within the canine metaphase spread. Wildtype shows co-localization of red (control) and green (experimental) probes. This co-localization is absent in the affected dog (homozygous for deletion), and is heterozygous in the carrier dog.

The presence of a deletion on *CFA14* was tested directly by FISH analysis. A long-range PCR product generated from the physical coordinates of failed SNPs served as the experimental, deletion-specific probe (14–63.6 g). A BAC-derived probe (14–59.1 or) was used as an internal control and for chromosome identification (the karyotype of the dog is complex, and most chromosomes are morphologically similar). The loss of LR-PCR 14–63.6 g signal from one chromosome in an obligate heterozygote and from both Chr 14 homologs in an affected dog (**Figure 3**) confirmed the presence of a deletion on the terminal end of *CFA14*. The deletion was observed in 100% of cells from the carrier and affected dogs, whereas the normal controls had no cells showing loss of this signal.

### Cloning the Physical Breakpoints of the Deletion

SNPs from the CanineHD SNP array are spaced approximately every 10 Kb. The breakpoint of the deletion presumably lay between a SNP that worked and one that did not, both at the proximal and the distal ends of the deletion. We reasoned that the location of breakpoints could be fine-mapped within each 10- to 20-kb interval based on the pattern of successful PCR amplification. Primer pairs were designed to produce amplicons of roughly equal size (500 bp) and approximately equal spacing (1 kb) across both intervals marking the edges of the deletion. Experimental design and results from amplicon walking are provided in **Figure S1**. Based on the pattern of successful and failed PCR amplification, we inferred the location of the breakpoints.

**Figure 4:**
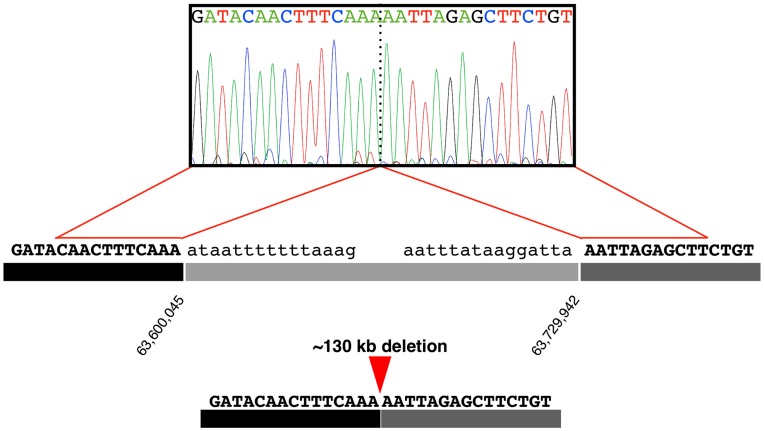
DNA sequence analysis revealing physical breakpoints. An electropherogram shows two sequences normally separated by 129,896 bp that have been brought adjacent by the deletion mutation.

A primer pair was selected to amplify across the proximal and distal breakpoints. Successful amplification from an affected dog would confirm the juxtaposition of two base pairs, which on a wild-type chromosome are separated by 50–150 kb. As expected, the PCR yielded product in affected dogs but failed in normal dogs. DNA sequencing of product from an affected dog established the actual breakpoint coordinates on *CFA14* (63,600,045 bp and 63,729,942 bp; **Figure 4**). Thus, 129,897 bp were deleted. The deletion abolished 14 of 15 exons of the *SLC13a1* gene (leaving only the first exon intact), translating into 95% of the peptide being lost. This suggested the mutation was a complete loss-of-function, null allele. We refer to the polymorphism as *Δslc13a1*.

### Δslc13a1 and Sulfate Metabolism

**Figure 5:**
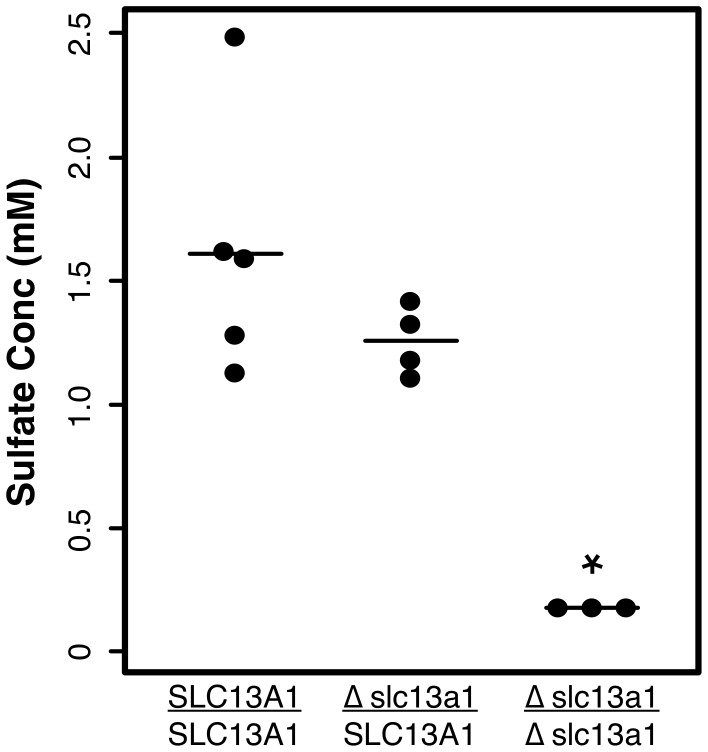
Serum sulfate levels by *SLC13A1* genotypic class. A scatter plot for sulfate concentration measurements from serum obtained from several dogs of each genotypic class: *SLC13A1* homozygotes (n = 5); heterozygotes (n = 4); and Δ*slc13a1* homozygotes (affected dogs; n = 3). The minimum sensitivity threshold for the assay is 0.2 mM. Differences between genotypes were assessed with the Kruskal Wallis test. Although sample sizes were relatively small, the mean sulfate level of affected dogs was significantly lower than the means of the other genotypic classes (*p* = 0.02).

Deletion of *SLC13A1* would be expected to cause decreased serum levels (hyposulfatemia) and increased urinary excretion (hypersulfaturia) of sulfate [10]. Serum and urine were recruited from dogs of each genotypic class to test these predictions. The results provided evidence of hyposulfatemia in affected dogs (**Figure 5** **)**. No sulfate was detected in the serum of three affected dogs. Results from measuring urinary sulfate were less conclusive owing to only a single affected dog tested. Nonetheless, of six dogs assayed, the affected dog had the highest excretion levels, and there was preliminary evidence of hypersulfaturia resulting from deletion of *SLC13A1*. The mean urinary sulfate levels in dogs with at least one copy of *Δslc13a1* were elevated relative to the mean level in wildtype Miniature Poodles (*p* = 0.02; **Table S3**).

### Developing a Co-dominant DNA Test

**Figure 6:**
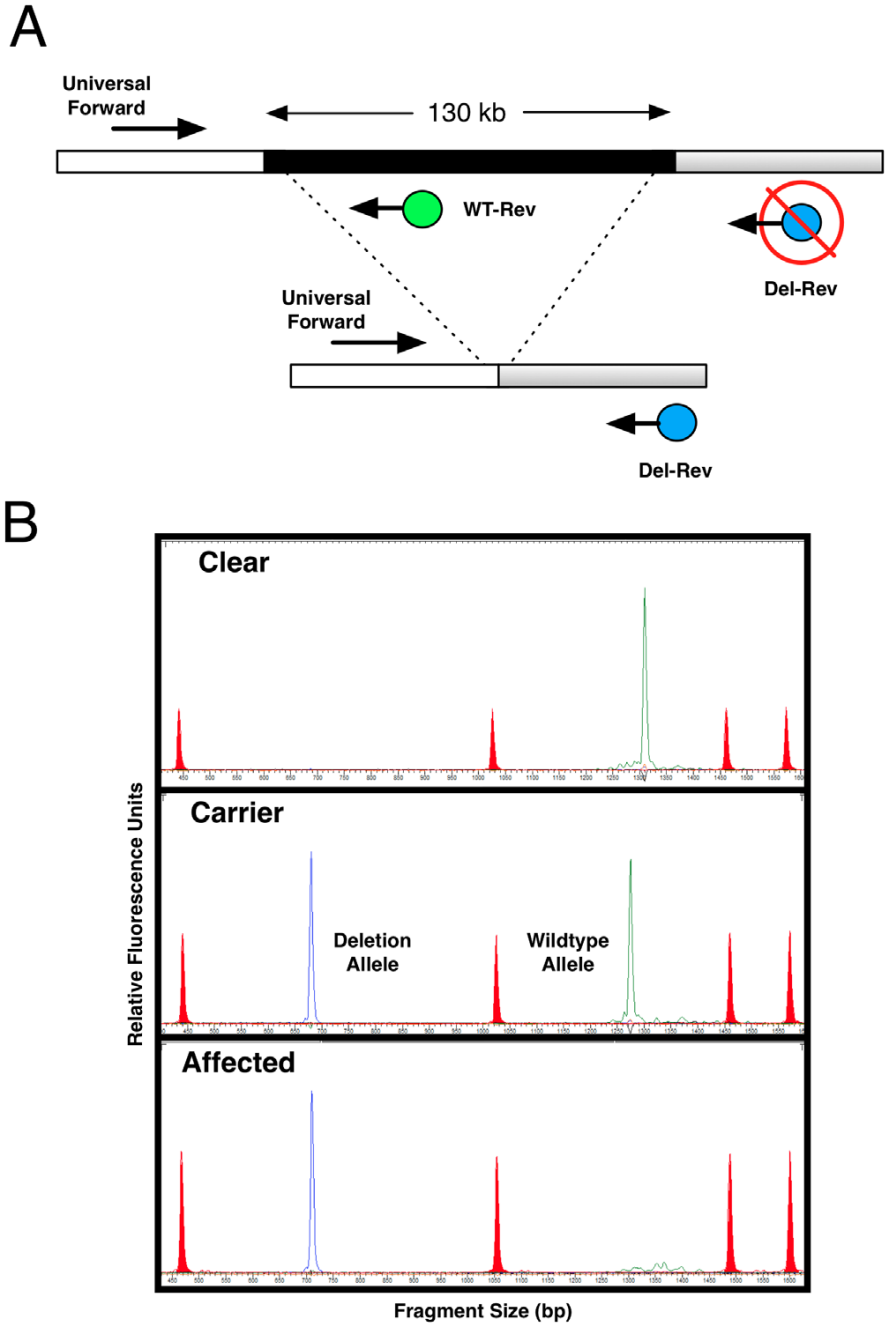
DNA test of large scale deletion. (A) A three-primer, dual reporter design exploits known breakpoints to create differentially dye-labeled and differentially-sized PCR products for a co-dominant DNA test readout. (B) The assay converted to a high-throughput format by analysis with a semi-automated DNA sequencing instrument for fluorescence-based fragment analysis. The electropherogram shows standard fragments for sizing (red peaks), product corresponding to the deletion-specific allele (blue peak), and product corresponding to the wildtype reference allele (green peak). Test outcomes are shown for each of the three genotypes possible. There is a size difference in the products corresponding to the wildtype allele in the clear dog and the carrier dog. This is attributable to a small insertion/deletion in the interval that segregates in the Miniature Poodle population.

Based on the physical breakpoint positions, we designed a three-primer assay to simultaneously report both mutant and normal alleles in a single PCR. As shown in (**Figure 6** **)**, the assay provided a co-dominant readout of the deletion polymorphism and thus distinguished each of the three genotypic classes that were possible: normal, carrier, and affected. We applied the assay to 11 affected dogs and 24 parents of affected offspring and found the deletion was perfectly associated (homozygous in all cases, heterozygous in all obligate heterozygotes).

### Population Genetics of *Δslc13a1*

The DNA test was used to estimate the frequency of the mutation in the U.S. subpopulation of the Miniature Poodle gene pool. Samples were collected non-specifically to reduce biased submission. Dogs related to cases in this study were removed in an attempt to minimize ascertainment bias (n = 13). Of 318 dogs sampled, 287 were homozygous for the normal allele (normal; 90.3%) and 31 were heterozygous for the deletion (carriers; 9.7%). No dogs were found homozygous for the deletion, as expected. The frequency of carriers in the population suggested that the mutation was not restricted to a few bloodlines. This was consistent with the previously reported international distribution of osteochondrodysplasia in the Miniature Poodle population [1]. The DNA test for the mutation is expected to be of use for the breeding community at large.

## Discussion

The osteochondrodysplasia studied here was first described in Great Britain over 56 years ago [1]. Subsequent articles have documented additional cases in the United States [2], [5], [6], Great Britain [4], [7], and Germany [3]. We have characterized the molecular basis of an osteochondrodysplasia in the Miniature Poodle breed, and we mapped the cause of this disorder to a single locus homozygous in all affected dogs. This result was consistent with previous pedigree analyses that suggested an autosomal recessive mode of inheritance. The mutation we identified was a large-scale deletion that removed all (14/15) but the first exon of *SLC13A1*. The deletion was found on an extended ancestral haplotype, indicating a singular origin, and was perfectly associated with the osteochondrododysplasia. No non-affected dogs were found to be homozygous for the deletion (*n* = 331), consistent with the disorder being Mendelian and fully penetrant.

A DNA-based test was developed that reliably provides information on both alleles (presence and absence of the deletion). This co-dominant test will enable breeders to avoid carrier-carrier crosses, thereby preventing litters with affected dogs in the near term. The test will ultimately allow the elimination of this deleterious mutation from the breed. A survey of Miniature Poodle dogs from the United States provided an allele frequency of 5%, suggesting a carrier frequency of approximately 10% (assuming HWE and no ascertainment biases in sampling). This frequency may differ among other geographic subpopulations and other varieties of Poodle. Reports of the disorder in European dogs 40–50 years ago suggest the mutation is now broadly distributed.

Mutation of *SLC13A1* implicates aberrant sulfate metabolism in the etiology of Miniature Poodle osteochondrodsyplasia. Sulfate is a hydrophilic anion that requires active transport across the membrane in all cells. There are two classes of specialized transporters–the sodium-dependent sulfate symporters, with two members, and the sodium-independent transporters, with eight members. *SLC13A1* is a sodium-dependent sulfate symporter that helps regulate serum levels of sulfate [13]. *SLC13A1* is expressed in the luminal epithelium of the small intestine, where it mediates absorption of dietary sulfate. *SLC13A1* is also expressed in the renal epithelium, where it mediates reabsorption from the proximal renal tubule of the kidney. Loss of murine *SLC13A1* has been shown to result in hyposulfatemia and hypersulfaturia [10].

We tested for hyposulfatemia and hypersulfaturia in affected Miniature Poodles by measuring circulating and excreted levels of sulfate in samples obtained from each *SLC13A1* genotypic class. The hypersulfaturia experiment was inconclusive because we were only able to recruit urine from a single affected dog. It is worth noting, however, that the affected dog showed the highest urinary level among seven dogs tested. Further experiments are needed to demonstrate that deletion of *SLC13A1* causes hypersulfaturia. Our results with serum sulfate levels were more conclusive. The average serum sulfate for healthy, non-affected dogs was 1.4±0.4 mM. In contrast, the levels in affected dogs were below the limit of detection for the assay (<0.2 mM). This observation was consistent with Δslc13a1 being a null allele.

*SLC13A1* does not appear to be expressed in cartilage or bone, indicating that the osteochondrodysplasia that results from deletion of *SLC13A1* is a metabolic disorder, likely impacting other systems and resulting in phenotypes that have not yet been described clinically. This prediction is strongly supported by the previous work of Dawson *et al*. with the mouse knockout model of *SLC13A1*. Mutant mice exhibited growth retardation similar to that observed in Miniature Poodles, but the murine skeletal defects were not as profound. Subsequent work in mice has shown several other phenotypes as well, such as lowered fertility and clonic seizures in early adulthood [10]. Subsequent work in the mouse model has shown gastrointestinal abnormalities [15], altered neurochemistry [16], impaired memory [17], decreased object-induced anxiety [18], decreased activity levels, and a possible increase in longevity [19]. None of the affected dogs in our study have suffered idiopathic seizures; other phenotypes have not yet been investigated [10].

Interestingly, the osteochondrodysplasia of Miniature Poodles more closely resembles the phenotypes associated with mutation in *SLC26A2*, a sodium-independent sulfate transporter with ubiquitous expression in human and mouse. Seven paralogs of *SLC26A2* are expressed in other tissues, but *SLC26A2* is the predominant sulfate transporter of cartilage and bone [20]. Sulfate metabolism in these tissues is important for sulfation of proteoglycans in the extracellular matrix (ECM). Specifically, sulfate is a key modification of glycosaminoglycans (GAGs), including chondroitin, dermatan, keratan, and heparan sulfate GAGs. GAGs are important post-translational modifications of a vast array of proteoglycans involved in many biological processes. In cartilage and bone, chondroitin sulfate is the key modification of aggregan, the most abundant proteoglycan in these tissues. The electrostatic repulsion from the dense, negative charges from sulfation provides resistance to compression, a key adaptation of cartilage.

Disruption of human *SLC26A2* causes a spectrum of osteochondrodysplasias, including achondrogenesis type 1B (OMIM#600972) [21], atelosteogenesis type II (OMIM#256050) [22], recessive multiple epiphyseal dysplasia (OMIM#226900) [23], [24], and diastrophic dysplasia (OMIM#222600) [25]. The shared features of these disorders are growth retardation, disproportionately short limbs, narrow, flattened chest, rounded abdomen, clubfoot, cleft palate, and “hitchhiker” thumbs (OMIM.org). This is relevant to the current study in that affected Miniature Poodles show the phenotypes of the *SLC26A2* human disease spectrum, consistent with shared mechanism of undersulfation of proteoglycans.

In conclusion, our results suggest that the hyposulfatemia and hypersulfaturia resulting from loss of *SLC13A1* function in affected Miniature Poodles manifests principally as an osteochondrodysplasia, but that the mutation is more likely responsible for a metabolic syndrome that has not yet been completely described, in dog or in human. None of the human osteochondrodysplasias have been tied to *SLC13A1* mutations, suggesting species-specific background effects that may either suppress or exacerbate *SLC13A1* loss-of-function. Such species-specific modifiers may also be responsible for differences in the phenotypic presentation between the mouse and canine models.

## Materials and Methods

### Ethics Statement

All samples were obtained with informed owner consent according to IACUC protocol #11-02-002 (Van Andel Research Institute).

### Subjects, Sample Recruitment, and DNA

In this study, affected cases and non-affected controls were purebred Miniature Poodles recruited by a liaison through an informal network of breeders and owners having unknown ascertainment biases. All cases were vetted clinically by an attending veterinarian. Cases were examined at different ages, but all had noticeably stunted growth, flared rib cages, and misshapen paws, consistent with prior clinical reports of the defect [1]–[7]. Blood or saliva samples were collected from cases and controls. DNA was prepared from the samples by standard methods of extraction, modified for integration with a semi-automated workflow (AutogenFlexStar, Holliston, MA). Samples were also collected with buccal swabs at dog show conformation events to establish a population cohort capable of representing the baseline of the mutation frequency. DNA was prepared from buccal cells as previously described [26].

### Whole Genome SNP Genotyping

Single nucleotide polymorphism (SNP) genotyping was performed with the CanineHD BeadChip array (Illumina, San Diego, CA) according to the manufacturer’s instructions. The platform is based on arrayed oligonucleotides, each corresponding to a known physical polymorphism in the dog genome, which can be assayed by enzymatic single-base extension and dual-color fluorescence to report bi-allelic calls. The array offers 173,662 SNP loci at an average density of 70 SNPs per megabase. Raw fluorescence intensity data generated from a BeadArray Reader were converted to curated genotypes with *GenomeStudio* software using the pre-set genotypic cluster algorithms provided by Illumina.

### Cell Culture for Metaphase FISH

Peripheral blood (3–5 ml) was obtained in sodium heparin blood collection tubes from two carriers, two affected, and four normal control Miniature Poodles. The buffy coat was removed and cultured in PB-MAX karyotyping medium (Invitrogen, Grand Island, NY) for 72 h. Cells were arrested in metaphase with the addition of 0.1 µg/ml of colcemid (Invitrogen, Grand Island, NY ) for 5 h and then were harvested by standard cytogenetic procedures for metaphase slide preparations.

### Fluorescence in situ Hybridization (FISH)

A control FISH probe (14–59.1or) was prepared from purified BAC DNA of clone CH82–134 K03 (BACPAC Resources Center). This BAC maps to Chr14; 59029668–59238895; UCSC Dog genome browser (Broad/canFam2) v245). A long-range (LR) PCR product was generated with primer pairs located within the deleted region (**Table S1**). LR-PCR was performed with a Herculase II Fusion kit (Agilent Technologies, Santa Clara, CA) according to the manufacturer’s instructions. Briefly, 100 ng of high molecular weight genomic DNA was used as template in a 30 cycle reaction with 4% DMSO and an annealing temperature of 60°C. The LR-PCR product was purified with a Qiaquick spin-column (Qiagen,Valencia, CA) and labeled for use as a deletion-specific FISH probe (LR-PCR 14–63.6 g). Both the BAC-derived DNA and the LR-PCR DNA were labeled by nick translation with Orange-dUTP and Green-dUTP (Abbott Laboratories, Abbott Park, IL), respectively. In addition to showing strong specificity to the deleted region, the LR-PCR 14–63.6 g FISH probe also produced background fluorescence in some centromeric regions. This background fluorescence did not confound cytogenetic analysis. Metaphase FISH slides were prepared and images were acquired as previously described [27]. Between 20–30 metaphases were scored concordantly for each sample.

### Cloning Deletion Breakpoints

Primer pairs were designed with PRIMER3 software to devise amplicons evenly spaced (1-kb increments) across intervals of interest (10–20 kb). The amplicons were designed to infer the presence or absence of DNA segments based upon the success or failure of PCRs performed in triplicate. Amplicons tested the presence of sequences between the last SNP that yielded genotypes in all cases and the first SNP that failed in all cases. On the proximal end, the interval was less than 10 kb and fell between BICF2S2304347 (63,599,238 bp) and BICF2P354264 (63,608,713 bp). On the distal end, the interval was less than 17 kb and fell between BICF2P894315 (63,721,709 bp) and BICF2S23034397 (63,739,098 bp). PCR products were assayed by conventional agarose gel electrophoresis. The primer sequences used, their physical location, and the expected amplicon sizes are listed in (**Table S2)**. Once physical breakpoints were localized on each side of the deletion, a pair of primers was selected to amplify across the presumed deletion (**Table S1**). This amplicon was sequenced to confirm the actual breakpoints of the deletion by comparison with the publicly available build of the Boxer genome (*canFam2*) as a reference. During the course of this work, a newer build (canFam3) became available. The coordinates for conversion to the new build are available as supporting information (**Document S2**).

### Sulfate Measurements

Measurement of inorganic sulfate was performed by *BioAssay Systems* (Hayward, CA) using the QuantiChrom Sulfate Assay (DSFT-200) as previously described by Lundquist et al [28]. This turbidimetric measurement at OD_600_ has a linear range of detection of 0.02–2.00 mM sulfate. For the serum assays, peripheral blood (3–5 ml) was obtained in no additive, serum collection tubes. Urine was collected via urinary catheter and free catch. Measurements were performed in duplicate with a linear range of four standards across the linear range analyzed concurrently, which gave a correlation coefficient of R^2^ = 0.995 [29].

### DNA Test Design

A three-primer assay designed to provide a co-dominant DNA report of the deletion genotype is shown schematically in **Figure 6**. A universal forward primer (**Table S1**) was designed against sequences upstream of the deletion near the proximal breakpoint boundary. A wild-type reverse primer (Wt-Rev) was designed downstream (315 bp) and labeled with the VIC fluorophore. A mutant reverse primer (Mut-Rev) was designed 110 kb downstream, just beyond the distal breakpoint, and labeled with the FAM fluorophore. The deletion removes the Wt-Rev primer binding site, which brings the Mut-Rev primer site to within 242 bp of the universal forward primer site, thereby enabling PCR amplification. **Figure 6** diagrams the possible outcomes obtained with this assay.

### Data Analysis

SNP genotype data were analyzed using PLINK [30]. The data were filtered to exclude i) individual dogs with call rates lower than 98%; ii) SNPs with minor allele frequency (MAF) <0.05; iii) SNPs with call rates less than 99.8%; and iv) SNPs that did not pass the test for Hardy–Weinberg equilibrium (HWE) in the control cohort (p<0.05). P-values were adjusted for multiple tests, and the genome-wide significance threshold was set by permutation of case-control labels (100,000 iterations). Allele and genotype frequencies for the deletion were obtained from direct counts of sampled Miniature Poodles. Differences in serum sulfate measures between genotypic classes were tested for statistical significance by the Kruskal-Wallis test [31]. This is a non-parametric alternative to one-way analysis of variance.

## Supporting Information

Figure S1 **Overview of experiment to localize breakpoints.** SNP markers on the fixed array are shown in order. Orange SNPs failed to yield genotypes on affected dogs. An internal set of three SNPs gave heterozygous calls in cases. PCR amplicons were designed to span the interval between the last SNP typed and the first SNP that failed. Blue amplicons were successfully amplified in affected dogs, whereas red amplicons failed. One amplicon (shown in gray) failed to yield product. All PCRs were performed in triplicate.
(TIFF)

Table S1 **Primers used in this study.**
(PDF)

Table S2 **Description of serial amplicons used to localize upstream and downstream breakpoints.**
(PDF)

Table S3 **Urinary sulfate levels and SLC13A1 genotype.**
(PDF)

Document S1 **Genes within the critical region mapped by GWAS.**
(DOC)

Document S2 **Reference build coordinates (canFam2 and canFam3).**
(DOC)
