# Supplementary figures and images for: Partial Deletion of the Sulfate Transporter SLC13A1 Is Associated with an Osteochondrodysplasia in the Miniature Poodle Breed
Source: PLoS One. 2012 Dec 26;7(12):e51917. doi: 10.1371/journal.pone.0051917 (PMC3530542; doi:10.1371/journal.pone.0051917)

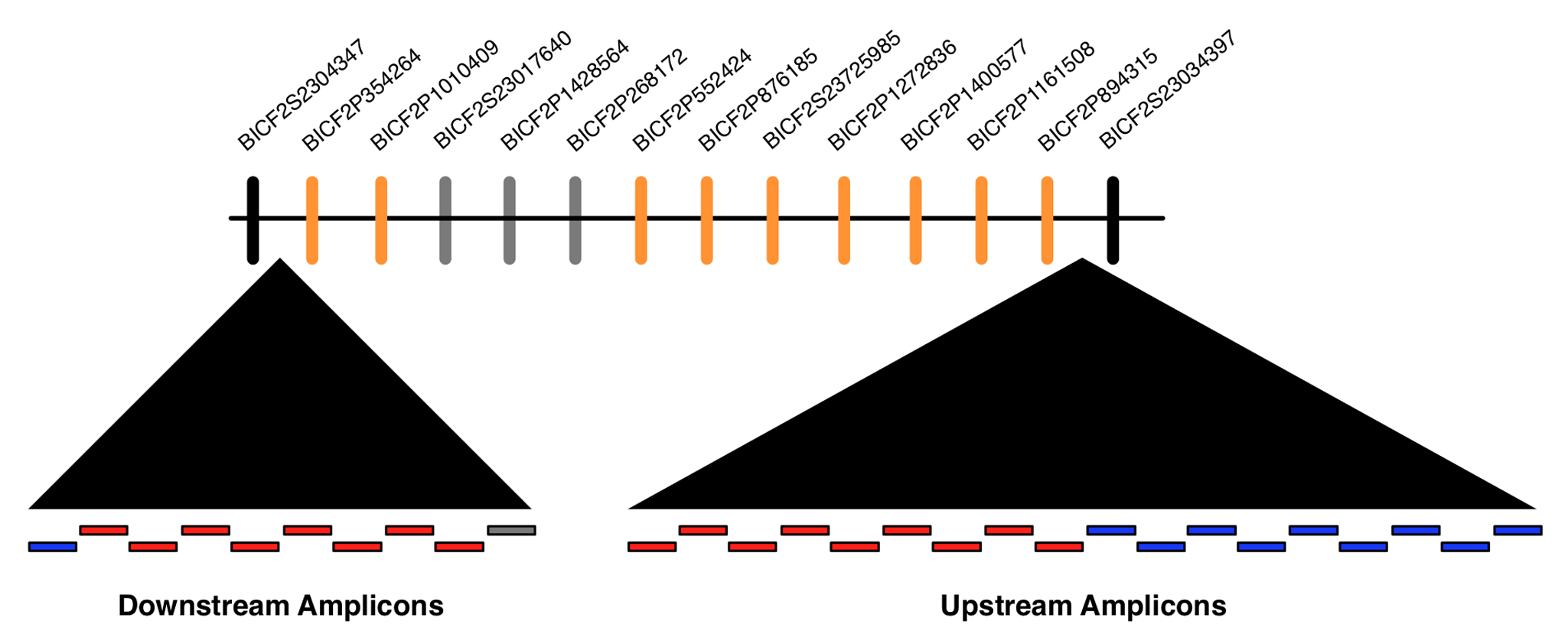

Supplement: Figure S1 — Overview of experiment to localize breakpoints. SNP markers on the fixed array are shown in order. Orange SNPs failed to yield genotypes on affected dogs. An internal set of three SNPs gave heterozygous calls in cases. PCR amplicons were designed to span the interval between the last SNP typed and the first SNP that failed. Blue amplicons were successfully amplified in affected dogs, whereas red amplicons failed. One amplicon (shown in gray) failed to yield product. All PCRs were performed in triplicate. (TIFF) [file pone.0051917.s001.tiff]
