# Supplementary material for: Partial Deletion of the Sulfate Transporter SLC13A1 Is Associated with an Osteochondrodysplasia in the Miniature Poodle Breed
Source: PLoS One. 2012 Dec 26;7(12):e51917. doi: 10.1371/journal.pone.0051917 (PMC3530542; doi:10.1371/journal.pone.0051917)
Supplement: Table S1 — Primers used in this study. (PDF) [file pone.0051917.s002.pdf]

Table S1. Supplemental information describing serial amplicons used to localize upstream and downstream breakpoints.

Amplicon	Forward Primer Sequence	Reverse Primer Sequence	Product (bp)	Chr	Start	Stop	AFF	NON
DEL_Upstream_10*	GAGACCTTCCCTTGGTTTCC	ACCAACACCCCTTCTGTCTG	480	14	63,599,279	63,599,758	+	+
DEL_Upstream_09	GACACAGGCAGAGGGAGAAG	AGCTGCCCACTCATTACCAC	474	14	63,600,103	63,600,576	-	+
DEL_Upstream_08	GGCAAAGGAGAGCAAAGAGA	GGTCACCTCACTTCATCAGTCA	522	14	63,600,754	63,601,275	-	+
DEL_Upstream_07	AACCCCTAGCTCTCCTGGAA	TATCTGCTTCCCCTGGACAC	496	14	63,601,372	63,601,867	-	+
DEL_Upstream_06	TTAGTGCCCCAGAGGTTACG	CAGCTCTCGGCAGCTCTC	492	14	63,602,008	63,602,499	-	+
DEL_Upstream_05	CAGGCCTCACCCACACAG	ACACCTCGTCGTGGTCCTC	450	14	63,602,981	63,603,430	-	+
DEL_Upstream_04	TGAGGACTGTGAGGACGTTG	TGTGTGGTGATGACATTTGCT	500	14	63,603,716	63,604,215	-	+
DEL_Upstream_03	AGTGAGGGGAGACGATTGTG	GGGTAATTTTCCCCTTTGGA	467	14	63,604,857	63,605,323	-	+
DEL_Upstream_02	ACCAGATGTCCCCACTGATG	ACTGGGCTCACAGGTCAGAG	483	14	63,605,606	63,606,088	-	+
DEL_Upstream_01**	GGAATTCCCTGCTGACTTGA	GAGTGCCTACTGCCCTTTGT	453	14	63,607,792	63,608,244	0	0
DEL_Downstream_01	TCTGAGTGGAAGAGGAGTCCA	AATTGCAGAGAGGAGCCAGA	400	14	63,722,082	63,722,481	-	+
DEL_Downstream_02	CCCACCACTTCCTATTGCTC	GGAGACCCAGGATTGAGTCG	461	14	63,722,714	63,723,174	-	+
DEL_Downstream_03	CTGGCCCTTAGTTCAGGTCA	GCAATTGCAATGCCTACCTC	407	14	63,723,560	63,723,966	-	+
DEL_Downstream_04	TCTCTTGCCAGGAAACAAGAA	CCATGATTGGTCCTGCTTTT	449	14	63,724,349	63,724,797	-	+
DEL_Downstream_05	ACACGGTGGCACCAGACA	CCCATCTTTCCCATCATCA	510	14	63,725,236	63,725,745	-	+
DEL_Downstream_06	AGGACCAGGGTGGAGGAT	TTCATGGTAGACAGGGAGAGG	508	14	63,726,965	63,727,472	-	+
DEL_Downstream_07	TTTGTTTAAATCACTTGGCACA	GAAAATGAAAATGAAGGGGATAGA	519	14	63,727,749	63,728,267	-	+
DEL_Downstream_08	GTGCACTCATGCTCTGCTGT	TGGCTTATCCCTGATTTGTCTT	449	14	63,728,744	63,729,192	-	+
DEL_Downstream_09	TCCTGATGTTTTAGTGTGAAGCA	TCTGTGCCATTTGCTGAAAG	519	14	63,729,669	63,730,187	-	+
DEL_Downstream_10*	CGACGTTTGTTGGGTCACAT	CAAGAATATGGGCCTTTGGA	516	14	63,730,511	63,731,026	+	+
DEL_Downstream_11	GCCTAAGCTTGCATCGACTT	CAGTTCCCTGTTGGAGAAGG	493	14	63,731,511	63,732,003	+	+
DEL_Downstream_12	CAGGTCCCTCAGCTTGTGTA	CCAAGGAGATCCTGGATTAGC	524	14	63,732,471	63,732,994	+	+
DEL_Downstream_13	ATTTCTCCAGCCCATCATCA	CTGGCTCAGTGCGTTATCTG	485	14	63,733,467	63,733,951	+	+
DEL_Downstream_14	AGCTGATGCTCTTGTGCTGA	GGAAAAGAAAGCAGAGTTCCA	479	14	63,734,418	63,734,896	+	+
DEL_Downstream_15	CAGAAATGAGGGGACAGTGG	TCCATGATTGCACATGGTGT	516	14	63,735,380	63,735,895	+	+
DEL_Downstream_16	GGATTGAACAAGACACGTGAAA	AGTACGCAGGGAGCCAACTA	499	14	63,736,329	63,736,827	+	+
DEL_Downstream_17	GGATCCCGAGGATGGTTTAT	GCAATGTGTGGCAGTTATGG	407	14	63,737,316	63,737,722	+	+
DEL_Downstream_18	ACTCCAGATTCCCATGCAAC	ACTGTTACGCAAGGGGATTG	516	14	63,738,042	63,738,557	+	+

Primer pairs defined amplicons used to localize deletion breakpoints based on pattern of amplification in affected (AFF) and nonaffected (NON) dogs scored as either amplifiable (+) or not amplifiable (-). All experiments were performed in triplicate.
*Primers used to clone actual breakpoints by amplifying product that spanned the deletion in a homozygous (affected) dog.
**Primer pair excluded because of failed PCRs in control samples.
The forward primer of DEL_Upstream_10 and the reverse primer of DEL_Downstream_10 yielded a 1.8 kb (Exp. 1,851) bp PCR product in a non-affected dog.
