# Supplementary material for: Partial Deletion of the Sulfate Transporter SLC13A1 Is Associated with an Osteochondrodysplasia in the Miniature Poodle Breed
Source: PLoS One. 2012 Dec 26;7(12):e51917. doi: 10.1371/journal.pone.0051917 (PMC3530542; doi:10.1371/journal.pone.0051917)
Supplement: Table S2 — Description of serial amplicons used to localize upstream and downstream breakpoints. (PDF) [file pone.0051917.s003.pdf]

Table S2. Additional primers used in this study.

Name	Primer Sequence	Start
DEL_Upstream_10F_T3*	aattaaccctcactaaagggaGAGACCTTCCCTTGGTTTCC	63,599,279
dnD-R_T7*	taatacgactcactatagggCACGTGGGCTTGTCAGAATA	63,730,120
WT_Rev-VIC**	CAGATGCTGATCAGAAACATCTAT	63,729,824
Mut_Rev-FAM**	TCCCCATGTAAGCTTTTCCA	63,599,981
Universal Forward**	CACGTGGGCTTGTCAGAATA	63,730,120
LR-Forward***	TTGTTTGATGTCTAGATTTGGCTTGCTTCATAGACC	63,662,018
LR-Reverse***	CTCCAGAGGCCTAAGAGCAACAACCAGAAATGAT	63,672,379

All primer sequences were from Chr 14.
Lowercase sequences denote artificial tags for fluorescence and sequencing.
*Primers used to clone and sequence breakpoints with T3 and T7 tags.
Breakpoint cloning yielded a 945 bp product.
**Primers used in three primer fluorescence-based DNA test.
***Primers used for long-range PCR; product was subsequently used as probe for FISH.
Long-range PCR yielded a 10,394 bp product.
