# Supplementary material for: Partial Deletion of the Sulfate Transporter SLC13A1 Is Associated with an Osteochondrodysplasia in the Miniature Poodle Breed
Source: PLoS One. 2012 Dec 26;7(12):e51917. doi: 10.1371/journal.pone.0051917 (PMC3530542; doi:10.1371/journal.pone.0051917)
Supplement: Table S3 — Urinary sulfate levels and SLC13A1 genotype. (PDF) [file pone.0051917.s004.pdf]

Table S3. Urinary sulfate levels and *SLC13A1* genotype.

Genotype	ΔOD_{600} 1	2	ΔOD_{600} (Avg.)	Sulfate (mM)
SLC13A1 / SLC13A1	0.283	0.280	0.282	51.5
SLC13A1 / SLC13A1	0.407	0.399	0.403	73.7
SLC13A1 / SLC13A1	0.379	0.377	0.378	69.2
Δslc13a1 / SLC13A1	0.519	0.511	0.515	94.2
Δslc13a1 / SLC13A1	0.551	0.549	0.550	100.7
Δslc13a1 / Δslc13a1	0.575	0.573	0.574	105.0

A heterozygous dog had an unusually low urinary sulfate measure (2.1 mM; 25-fold below any other measure). We interpreted this as a technical failure and excluded the sample from further analysis.

Insufficient sample size precluded testing for hypersulfaturia in affected dogs specifically. However, a difference exists in the mean urinary sulfate levels between wildtype dogs (64.8 ± 11.7 mM) and dogs with at least one copy of the deletion (100.0 ± 5.4 mM). This difference is statistically significant ($p = 0.02$; two-tailed t-test).
