# Supplementary material for: Partial Deletion of the Sulfate Transporter SLC13A1 Is Associated with an Osteochondrodysplasia in the Miniature Poodle Breed
Source: PLoS One. 2012 Dec 26;7(12):e51917. doi: 10.1371/journal.pone.0051917 (PMC3530542; doi:10.1371/journal.pone.0051917)
Supplement: Document S1 — Genes within the critical region mapped by GWAS. (DOC) [file pone.0051917.s005.doc]

**Document S1** – **Genes within the critical region mapped by GWAS:**

The core haplotype common to all cases extends Chr14:62,747,406-63,938,239 (canFam2). This encompasses a total of 1.19 Mb. The interval contains six genes:

***PTPRZ1***, Receptor-type tyrosine-protein phosphatase zeta; phosphacan

***AASS***, Mitochondrial alpha-aminoadipic semialdehyde synthase

***CADPS2***, Calcium-dependent secretion activator 2

***FEF1***, Fez family zinc finger 1, transcription repressor; regulator of axonal projections

***SLC13A1***, sodium/sulfate symporter

***IQUP***, IQ motif and ubiquitin domain-containing transcript
