# Supplementary material for: Partial Deletion of the Sulfate Transporter SLC13A1 Is Associated with an Osteochondrodysplasia in the Miniature Poodle Breed
Source: PLoS One. 2012 Dec 26;7(12):e51917. doi: 10.1371/journal.pone.0051917 (PMC3530542; doi:10.1371/journal.pone.0051917)
Supplement: Document S2 — Reference build coordinates (canFam2 and canFam3). (DOC) [file pone.0051917.s006.doc]

**Document S2** – **Reference build coordinates (canFam2 and canFam3):**

During the course of this work, a new reference build (canFam3) became publicly available. Below are the coordinates for key mapping attributes for both canFam2 and canFam3:

Critical region (conserved ancestral haplotype block):

canFam 2 = Chr14:62,747,406-63,938,239 (1,190,833 bp)

canFam 3 = Chr14:59,779,966-60,966,679 (1,186,713 bp)

Deletion breakpoints:

canFam2 = Chr14:63,600,045-63,729,942 (129,897 bp)

canFam3 = Chr14:60,628,771-60,758,561 (129,790 bp)
